# Enhanced Oral Efficacy of Semaglutide via an Ionic Nanocomplex with Organometallic Phyllosilicate in Type 2 Diabetic Rats

**DOI:** 10.3390/pharmaceutics16070886

**Published:** 2024-06-30

**Authors:** Gyu Lin Kim, Jae Geun Song, Hyo-Kyung Han

**Affiliations:** College of Pharmacy, Dongguk University-Seoul, Dongguk-ro-32, Ilsan-Donggu, Goyang 10326, Republic of Korea

**Keywords:** semaglutide, oral formulation, colonic delivery system, obesity, diabetes

## Abstract

This study aimed to develop an effective oral formulation of semaglutide, a glucagon-like peptide-1 receptor agonist, using an organometallic phyllosilicate-based colonic delivery system. The core nanocomplex (AMP-Sema) of 3-aminopropyl-functionalized magnesium phyllosilicate (AMP) and semaglutide was prepared via electrostatic interactions. Subsequently, AMP-Sema was coated with a polymer showing pH-dependent solubility (Eudragit^®^ S100) for preferential colonic delivery. The surface-coated nanoparticles (EAMP-Sema) showed a narrow size distribution, and the encapsulated semaglutide maintained its conformational stability. The pH-dependent drug release property of EAMP-Sema yielded around 20% and 62% drug release at pH 1.2 and 7.4, respectively. The nanoparticles exhibited significantly decreased size and surface charge at pH 7.4, which indicated the pH-dependent dissolution of the coating layer. Furthermore, EAMP-Sema effectively improved the membrane permeability and metabolic stability of semaglutide in the gastrointestinal tract. It protected the encapsulated drugs from proteolysis in simulated intestinal fluids and increased drug transport by 2.5-fold in Caco-2 cells. Consequently, orally administered EAMP-Sema (equivalent to 8 mg/kg of semaglutide) showed significant therapeutic benefits, yielding effective glycemic control and weight loss in high-fat diet/streptozotocin (40 mg/kg)-induced type 2 diabetic rats. These results demonstrate that EAMP-Sema could improve the efficacy of orally administered semaglutide by enhancing the GI stability and cellular uptake of protein drugs.

## 1. Introduction

Protein drugs have notable therapeutic potential for the treatment of various diseases, and this is demonstrated by their continuously increasing global market shares and clinical values [1,2]. However, protein drugs are vulnerable to certain environmental and biological stresses, leading to the loss of their activities during the manufacturing process and storage and in the body upon administration. Furthermore, their high molecular weight and hydrophilicity contribute to a low membrane permeability [3,4]. Consequently, orally administered protein drugs face multiple barriers and issues, despite oral administration being the most preferred and non-invasive route of administration for many patients. Various approaches have been attempted to enhance the efficacy of orally administered protein drugs. For example, absorption enhancers have been used to improve drug absorption by various mechanisms: changing the membrane fluidity or mucus viscosity, opening tight junctions, and facilitating drug diffusion across the intestinal membrane [5,6,7,8]. Enteric coating technology and various drug delivery systems, such as liposomes, microemulsions, hydrogels, and nanoparticulate carrier systems, have also been utilized to improve the gastrointestinal (GI) stability and membrane permeability of protein drugs [9,10,11,12]. Although these approaches achieved some level of success, there are still many hurdles to overcome for efficient oral delivery of protein drugs.

Semaglutide is a glucagon-like peptide-1 (GLP-1) receptor agonist displaying 94% structural homology with human GLP-1 [13]. It is used for the treatment of type 2 diabetes and as an anti-obesity medication. Semaglutide is a fatty-acylated GLP-1 analogue showing increased resistance to dipeptidyl peptidase-4, and it offers several advantages over insulin: (i) low risk of hypoglycemia, (ii) long half-life (~168 h), and (iii) suppression of appetite and consequent decrease in food intake. The administration route currently employed for semaglutide in the clinic is either subcutaneous injection once a week or oral administration daily. Considering the long-term treatment durations of diabetes and obesity, oral administration is more desirable than injection. A recently approved oral formulation of semaglutide uses sodium N-(8-[2-hydroxybenzoyl]amino)caprylate (SNAC) as an absorption enhancer [14]. However, this formulation requires fasting (no food and no drinking) for 30 min after administration, which may be inconvenient for patients [15,16,17]. In addition, SNAC may cause gastrointestinal side effects, such as nausea and diarrhea [18,19,20,21].

Among various types of organoclays, 3-aminopropyl-functionalized magnesium phyllosilicate (AMP) may be a good alternative to SNAC for enhancing the oral absorption of semaglutide [22]. As a cationic nanoparticle, it can easily form an ionic nanocomplex with negatively charged drugs in aqueous conditions. The AMP-based ionic nanocomplexes offer several advantages for protein drugs. First, they can enhance the cellular uptake of protein drugs by facilitating endocytosis and paracellular transport [23,24]. Previous studies demonstrated that AMP has a transient tight junction opening effect and thus increases the paracellular drug uptake [23,24]. In addition, AMP promotes the cellular uptake of proteins via endocytosis [24]. Therefore, the formation of an AMP-based nanocomplex may enhance both transcellular and paracellular drug uptake. Given that AMP can interact with a structurally diverse range of protein drugs, it is broadly applicable as a drug delivery carrier. Furthermore, a pH-responsive surface coating of AMP-based nanocomplex may provide additional advantages in improving the oral absorption of protein drugs, since it can minimize drug exposure in the stomach and deliver drugs to the colon, which has low proteolytic activity, neutral pH, and high sensitivity to absorption enhancers [25].

In this study, an AMP-based ionic nanocomplex was fabricated as an oral formulation of semaglutide and was surface-coated with a pH-dependent polymer (Eudragit S100) for colonic delivery. The obtained formulation was systematically characterized in vitro and subjected to in vivo efficacy studies using type 2 diabetes rat models.

## 2. Materials and Methods

### 2.1. Materials

Semaglutide was purchased from Hangzhou Peptide Biotechnology Co., Ltd. (Hangzhou, China). Streptozotocin (STZ) and digestive enzymes (pepsin, trypsin) were obtained from Sigma-Aldrich Co. (St. Louis, MO, USA). Eudragit ^®^ S100 (poly(methacrylic acid-co-methyl methacrylate) (1:2)) was obtained from Evonik Korea Ltd. (Seoul, Republic of Korea). Inorganic salts were purchased from Junsei Chemical Co., Ltd. (Tokyo, Japan). AMP was synthesized as described in previous studies [26,27]. HPLC-grade solvents (acetonitrile, methanol, and all other organic solvents) were purchased from Avantor Co. (Radnor, PA, USA). Dulbecco’s Modified Eagle’s medium (DMEM), fetal bovine serum (FBS), antibiotics, and all other reagents used in the cell culture studies were purchased from GE Healthcare Life Sciences (South Logan, UT, USA).

Caco-2 cells (human epithelial colorectal adenocarcinoma) were obtained from the Korean Cell Line Bank (Seoul, Republic of Korea). Cells were cultured at 37 °C under 5% CO_2_ and 90% relative humidity, using DMEM supplemented with 10% FBS, 1% non-essential amino acids, and 1% antibiotics.

### 2.2. Preparation of AMP-Based Nanoparticles

AMP (30 mg) was dispersed in distilled water (3 mL) and sonicated for 10 min to obtain a clear solution. Semaglutide (10 mg/mL in distilled water, 1 mL) was added dropwise into the AMP clay solution (10 mg/mL, 3 mL) at room temperature, which immediately resulted in the formation of white precipitates. The mixture was then stirred at 300 rpm for 1 h and centrifuged (Combi R515, Hanil Scientific Inc., Gimpo, Republic of Korea) at 22,250× *g* and 4 °C for 15 min to collect the white precipitates. The collected nanoparticles (AMP-Sema) were dried under vacuum at room temperature.

For the surface coating, AMP-Sema (20 mg) dispersed in citric acid (10 mmol/L, 1 mL, pH 3) was added dropwise into a solution of 1% Eudragit^®^ S100 (2 mL) dissolved in a mixture of ethanol and acetone (1:2, v/v). After stirring at 350 rpm for 0.5 h, the resultant nanoparticles (EAMP-Sema) were collected by centrifugation (22,250× *g*, 4 °C, 15 min) and dried under vacuum at room temperature.

### 2.3. In Vitro Characterization

The hydrodynamic sizes, polydispersity indices (PDI), and zeta potentials of the nanoparticles (1 mg/mL) were determined using a Zetasizer (Nano-ZS90, Malvern, UK). The entrapment efficiency (EE, %) was calculated by using the following equation: (initial drug amount—drug amount in supernatant)/(initial drug amount) × 100.

Circular dichroism (CD) analysis with a Chirascan^TM^-Plus Spectrometer (Applied Photophysics, Surrey, UK) determined the secondary structure of semaglutide in the developed nanoparticles (1 mg/mL). The CD spectra were acquired in the wavelength of 200–260 nm, where the bandwidth and light path length were 1 nm and 0.5 mm, respectively. Fourier-transform infrared (FT-IR) spectroscopy of the nanoparticles (1 mg) was conducted in the wavelength range of 4000–500 cm^−1^, using an FT-IR spectrometer equipped with a ZnSe crystal accessory (Nicolet^TM^ iS^TM^5; Thermo Fisher Scientific Inc., Waltham, MA, USA). Transmission electron microscopy (TEM) was conducted at the National Center for Inter-University Research Facilities (Seoul National University, Seoul, Republic of Korea).

### 2.4. In Vitro Drug Release Studies

The drug release characteristics of the AMP-based nanoparticles (AMP-Sema and EAMP-Sema) were evaluated at pH 1.2 and 7.4 in a temperature-controlled water bath shaker (Biofree, Seoul, Republic of Korea). The nanoparticles (amount equivalent to 10 mg of semaglutide) were incubated in a hydrochloride buffer (100 mL of 0.1 mol/L solution at pH 1.2) or a phosphate buffer (100 mL of 0.05 mol/L solution at pH 7.4) while agitating at 100 rpm at 37 °C in a shaking water bath. The samples (0.1 mL) were collected from the solution at pre-determined time points, and an equal volume of fresh medium was replenished to maintain a constant volume of drug release medium. The collected samples were centrifuged at 22,250× *g* (4 °C, 15 min), and the drug concentrations in the supernatants were determined using high-performance liquid chromatography (HPLC) as described in Section 2.8. The hydrodynamic size distributions and zeta potentials of the nanoparticles were also monitored during the drug release study.

### 2.5. Gastrointestinal Stability

The gastrointestinal stability of the fabricated nanoparticles was evaluated using simulated gastric fluid (SGF) and intestinal fluid (SIF) containing pepsin (5 µg/mL) and trypsin (20 µg/mL), respectively [23]. The pH of the fluids was adjusted to pH 1.2 for SGF and pH 7.4 for SIF. The nanoparticles (3 mg) were added to either SGF (1 mL) or SIF (1 mL) while stirring at 100 rpm at 37 °C. After incubation for 4 h, 0.2 mol/L NaOH (0.2 mL) and 0.1 mol/L HCl (0.2 mL) were added into the SGF and SIF, respectively, to quench the enzymatic reactions. Following the centrifugation (22,250× *g*, 4 °C) for 15 min, the collected nanoparticles were incubated in a pH 7.4 phosphate buffer for 2 h. The conformational stability of the semaglutide released from the nanoparticles was examined using CD spectroscopy.

### 2.6. Transport Studies

Caco-2 cells were seeded at a density of 2.0 × 10^5^ cells/well in trans-well plates (12 wells with an insert membrane growth area of 1.12 cm^2^). The cells were then cultured at 37 °C for 3 weeks. Trans-epithelial electrical resistance (TEER) values were regularly measured using a Millicell ERS-2 epithelial tissue voltohmmeter (Merck KGaA, Darmstadt, Germany). After 3 weeks of culture, the medium was removed, and the cell monolayers were washed with HBSS twice. Next, HBSS was added to the apical (0.5 mL) and basolateral compartments (1.5 mL), which were equilibrated at 37 °C for 30 min. At the end of preincubation, the HBSS in the apical compartment was replaced with the nanoparticles (amount equivalent to 200 µg/mL of semaglutide) dispersed in the HBSS. At each time point, 150 µL of the sample was withdrawn from the basolateral compartment for analysis. After every collection, the compartment was replenished with an equal volume of fresh HBSS. The drug concentrations in the collected samples were determined using HPLC. TEER values were measured in the presence of the drug solution, and they were also measured after replacing the drug solution with fresh HBSS at the end of the transport studies.

The apparent permeability coefficient (P_app_) was assessed using the following equation: P_app_ = dQ/dt × 1/AC_0_, in which C_0_ represents the initial drug concentration in the apical compartment, A represents the surface area of the cell monolayer, and dQ/dt represents the rate of drug permeation across cells.

### 2.7. In Vivo Efficacy Studies

Animal studies were conducted following a study protocol approved by the Institutional Animal Care and Use Committee of Dongguk University (IACUC-2023-040-1). Male Sprague-Dawley rats (160–180 g) were subjected to a high-fat diet (HFD) for three weeks. On day 21, streptozotocin (STZ) dissolved in 50 mmol/L citrate buffer (pH 4.5) was intraperitoneally injected into rats at a dose of 40 mg/kg. After 10 days, their blood glucose concentrations were determined using a blood glucose meter (ACCU-CHEK^®^ guide, Roche, Basel, Switzerland) [28]. Rats with blood glucose concentrations over 300 mg/dL were considered to have STZ-induced type 2 diabetes and were used to assess the therapeutic efficacy of the semaglutide-loaded nanoparticles.

The STZ-induced type 2 diabetic rats were divided into four groups (*n* = 6 per group). Groups 1 and 2 were administered saline and semaglutide (0.4 mg/kg), respectively, once a week via subcutaneous injections. Groups 3 and 4 were orally administered a semaglutide solution and EAMP-Sema, respectively, once a day at equivalent doses to 8 mg/kg semaglutide. Every day, the rats were provided with a pre-determined amount of HFD and normal tap water. Various physiological parameters, including blood glucose, triglyceride (TG), total cholesterol (TC), glycated hemoglobin (HbA1c), body weight, and food/water intake, were continuously monitored over the entire treatment period (30 days). Prior to blood sample collection, rats were anesthetized using 1.5% isoflurane (Hana Pharmaceutical, Seoul, Republic of Korea) in O_2_ in a Tabletop Anesthesia System (Harvard Apparatus, MA, USA). Blood samples (0.2 mL) were collected from the jugular vein at the designated time points to monitor the concentration change in blood glucose, TG, TC, and HbA1c. Blood glucose concentrations and HbA1c levels were determined using a blood glucose meter (ACCU-CHEK^®^ guide) and a glycated hemoglobin meter (A1C EZ 2.0^TM^, BioHermes, Wuxi, China), respectively. TC and TG levels were measured using a Barozen lipid meter (Handok, Seoul, Republic of Korea).

### 2.8. HPLC Analysis

Drug concentrations in the samples collected from the in vitro drug release studies and transport studies were quantified by HPLC analysis. An Ultimate 3000 HPLC system (Thermo-Fisher) equipped with a reversed-phase column (C18, 4.6×150 mm, 5 µm) was used for the analysis. The column temperature was set at 40 °C. The following mobile phases were eluted: (A) 0.1% trifluoroacetic acid (TFA) in acetonitrile and (B) 0.1% TFA in water. The separation was achieved with a gradient elution of the mobile phases at a flow rate of 0.6 mL/min. The detection wavelength was set at 220 nm. The ratio of mobile phase B was sequentially changed from 60% to 50% from 0 to 2.5 min, to 40% from 2.5 to 3 min, maintained until 4 min, back up to 50% from 4 to 6 min, to 60% from 6 to 7 min, and maintained until 10 min. Tolbutamide was used as the internal standard. A linear calibration curve was constructed in the drug concentration range of 5 to 200 µg/mL (r^2^ > 0.999).

### 2.9. Statistical Analysis

All mean values are presented with their standard deviation (mean ± SD). Data normality was assessed using the Shapiro–Wilk test (IBM SPSS Statistics 29.0, International Business Machines Corporation, NY, USA), indicating *p*-values greater than 0.05. The Q-Q plots also confirmed data normality graphically. Subsequently, statistical analysis was performed using one-way ANOVA followed by Dunnett’s test. Statistical significance was set at a *p*-value < 0.05.

## 3. Results and Discussion

### 3.1. Preparation of AMP-Based Nanoparticles

AMP-Sema was fabricated by electrostatic interactions between negatively charged semaglutide and positively charged AMP, which led to a high entrapment efficiency of over 90%. As determined by dynamic light scattering (DLS) analysis, the prepared nanocomplexes showed an average size of 162 ± 3.63 nm and a zeta potential of −14.7 ± 0.35 mV (Table 1).

**Figure 1:**
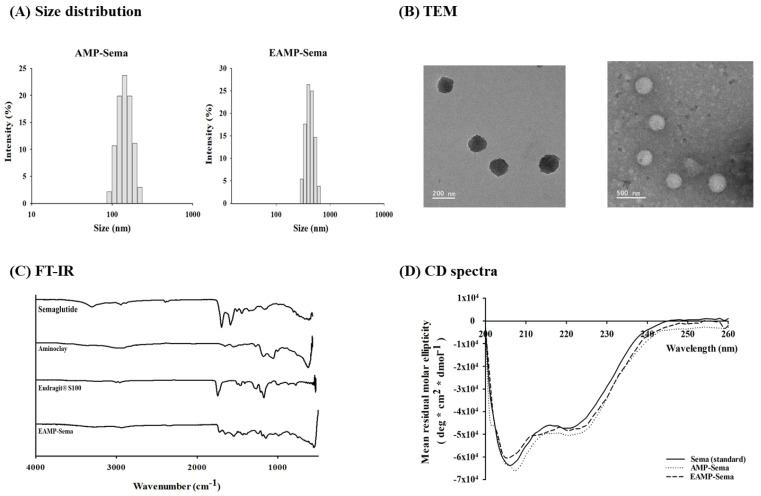
In vitro characterizations of AMP-based nanoparticles. (**A**) Hydrodynamic size distributions, (**B**) TEM images, (**C**) FT-IR spectra, and (**D**) CD spectra of semaglutide released from the nanoparticles at pH 7.4.

As shown in Figure 1A,B, the nanoparticles showed a spherical morphology with a narrow size distribution. Upon surface charge reversal of AMP-Sema in an acidic condition at pH 3, the nanoparticles were coated with Eudragit^®^ S100, yielding EAMP-Sema, to modulate the drug release at pH > 7.0. This surface coating resulted in significantly (*p* < 0.05) increased particle sizes (average size of 318 ± 1.23 nm for EAMP-Sema) (Table 1). Similar to AMP-Sema, EAMP-Sema also showed a spherical morphology with a narrow size distribution (Figure 1A,B). The TEM analysis confirmed the particle sizes determined by DLS analysis.

FT-IR spectroscopic analysis of EAMP-Sema exhibited the characteristic absorption bands of all components included in the formulation: semaglutide at 1652 cm^−1^ for amide Ⅰ and 1541 cm^−1^ for amide II; AMP at 990 cm^−1^ for Si-O-Si and 554 cm^−1^ for Mg-O; and Eudragit^®^ S100 at 1724 cm^−1^ for C=O ester and 1149 cm^−1^ for C-O ester (Figure 1C). In addition, the CD analysis indicated the conformational stability of semaglutide entrapped in AMP-based nanoparticles. As shown in Figure 1D, the CD spectra of semaglutide released from both AMP-Sema and EAMP-Sema were superimposed with that of the semaglutide standard. The results indicated that the inherent conformation of semaglutide could be retained even after the complexation.

Taken together, it was concluded that AMP-based nanoparticles were successfully fabricated with stably loaded semaglutide.

### 3.2. In Vitro Drug Release from AMP-Based Nanoparticles

**Figure 2:**
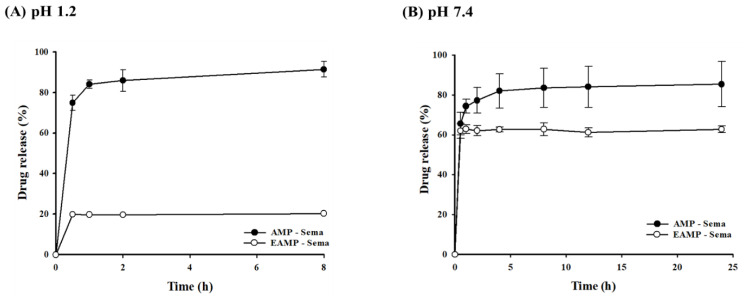
In vitro drug release of AMP-Sema and EAMP-Sema at (**A**) pH 1.2 and (**B**) pH 7.4 (mean ± SD, *n* = 3).

**Figure 3:**
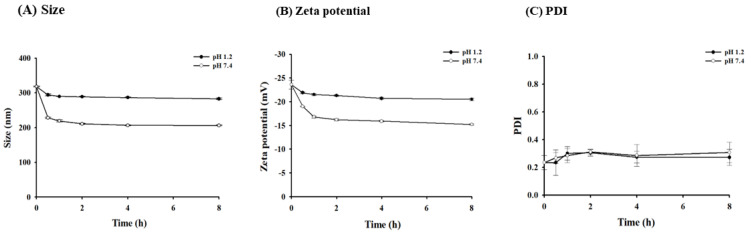
Changes in the size and surface charge of nanoparticles after incubating EAMP-Sema in the dissolution medium (mean ± SD, *n* = 3).

In both acidic and neutral conditions, the uncoated AMP-Sema displayed rapid drug release (Figure 2). Around 75% and 66% of the encapsulated drugs were released from AMP-Sema within 30 min at pH 1.2 and 7.4, respectively. In contrast, the coated EAMP-Sema showed a pH-dependent release rate. Around 20% and 62% of the encapsulated drugs were released from EAMP-Sema within 30 min at pH 1.2 and 7.4, respectively (Figure 2). This pH-dependent drug release property of EAMP-Sema was attributed to the surface-coated polymer’s solubility at pH 7.4. Accordingly, as shown in Figure 3, the size and surface charge of EAMP-Sema remained stable at pH 1.2, while undergoing significant changes at pH 7.4. These results are in accordance with previous findings [29,30,31,32]. For example, Tsai et al. [30] fabricated hyaluronan–cisplatin conjugate nanoparticles embedded in Eudragit S100-coated pectin/alginate microbeads, demonstrating that a Eudragit S100 coating suppressed drug release in acidic conditions while facilitating drug release at pH 7.4. In addition, previous studies [31,32] reported that the dissolution of Eudragit S100 at pH 7.4 led to a particle size reduction and alteration in the zeta potential.

Collectively, these results suggest that EAMP-Sema may minimize drug release in the acidic conditions of the stomach, thereby mediating a more preferential release of the drug in the lower intestine, where metabolic enzyme activities are relatively low [33].

### 3.3. Stability in Simulated Gastric and Intestinal Fluids

**Figure 4:**
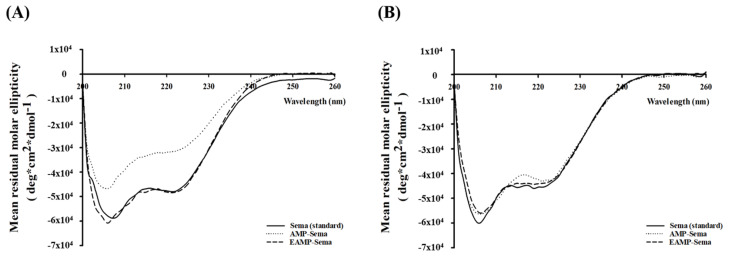
CD spectra of semaglutide released from AMP-based nanoparticles after the incubation in (**A**) SGF and (**B**) SIF. After incubating for 4 h in SGF or SIF, the nanoparticles were centrifuged and collected. These nanoparticles were then incubated in pH 7.4 PBS for 2 h. The released semaglutide was subjected to CD spectroscopic analysis.

The GI stability of semaglutide entrapped in AMP-Sema and EAMP-Sema was evaluated by incubating the nanoparticles in SGF and SIF with proteases [34]. In contrast to AMP-Sema, EAMP-Sema effectively protected the encapsulated semaglutide from chemical and enzymatic destabilization in SGF, maintaining the conformational stability of semaglutide. HPLC analysis also confirmed the presence of an intact drug peak without any additional impurity peaks (Figure S1). Surface coating with a pH-dependent polymer could minimize the exposure of encapsulated drugs to the harsh gastric environment, thus preventing the chemical and enzymatic degradation of drugs [35]. In SIF, semaglutide released from both types of AMP-based nanoparticles exhibited good conformational stability (Figure 4B). These results demonstrated the importance of delayed drug release in the GI tract for a more efficient drug delivery to the lower intestine, which has relatively less enzymatic activity and a milder pH than the stomach.

### 3.4. Transport Study

**Figure 5:**
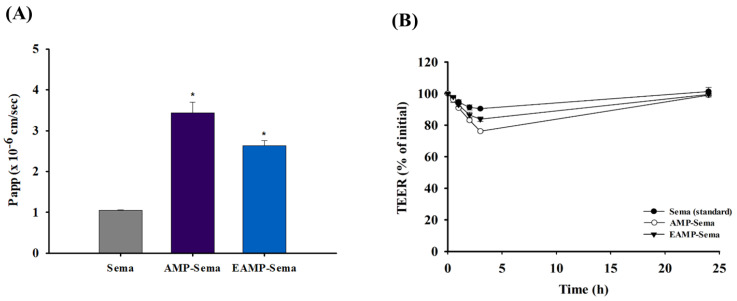
The impact of AMP-based nanoparticles on (**A**) the apparent permeability of semaglutide and (**B**) TEER values measured in Caco-2 cells (mean ± SD, *n* = 4). *: *p* < 0.05, compared to the control (free semaglutide).

The membrane permeability of the nanoparticles was assessed in Caco-2 cells. As summarized in Figure 5A, both AMP-Sema and EAMP-Sema significantly increased the drug permeability across the cell membrane, achieving 3.3-fold and 2.5-fold higher permeability compared to free semaglutide, respectively. In addition, both types of nanoparticles could induce a transient and reversible opening of tight junctions in Caco-2 cells (Figure 5B). In accordance with observations in drug release studies (Figure 3), the size and zeta potential of EAMP-Sema significantly decreased during the incubation in HBSS, implying the dissolution of the outer coating layer at pH 7.4 (Figure S2). Therefore, it is reasonable to expect that both EAMP-Sema and AMP-Sema would display comparable levels of tight junction opening effects. This effect could contribute to the enhancement of the cellular uptake of AMP-based nanoparticles. In addition to the paracellular pathway, AMP-based nanoparticles could facilitate drug uptake via endocytosis. The positively charged amine groups in AMP-Sema could interact with the negatively charged cell membrane, thereby enhancing endocytosis. The mechanism for the preferential uptake of cationic nanoparticles has not yet been clearly defined. However, it is hypothesized that the nanoparticles’ interaction with the negatively charged heparan sulfate proteoglycan groups located on the outer surface of cell membranes facilitates their adhesion to the membranes, thereby promoting the endocytic process [36]. Our previous studies also demonstrated that AMP-based cationic nanoparticles could facilitate cellular drug uptake via clathrin-mediated endocytosis [24]. Overall, AMP-based nanoparticles could significantly increase the cellular uptake of semaglutide, implying their potential for improving oral drug delivery.

### 3.5. In Vivo Efficacy Studies

**Figure 6:**
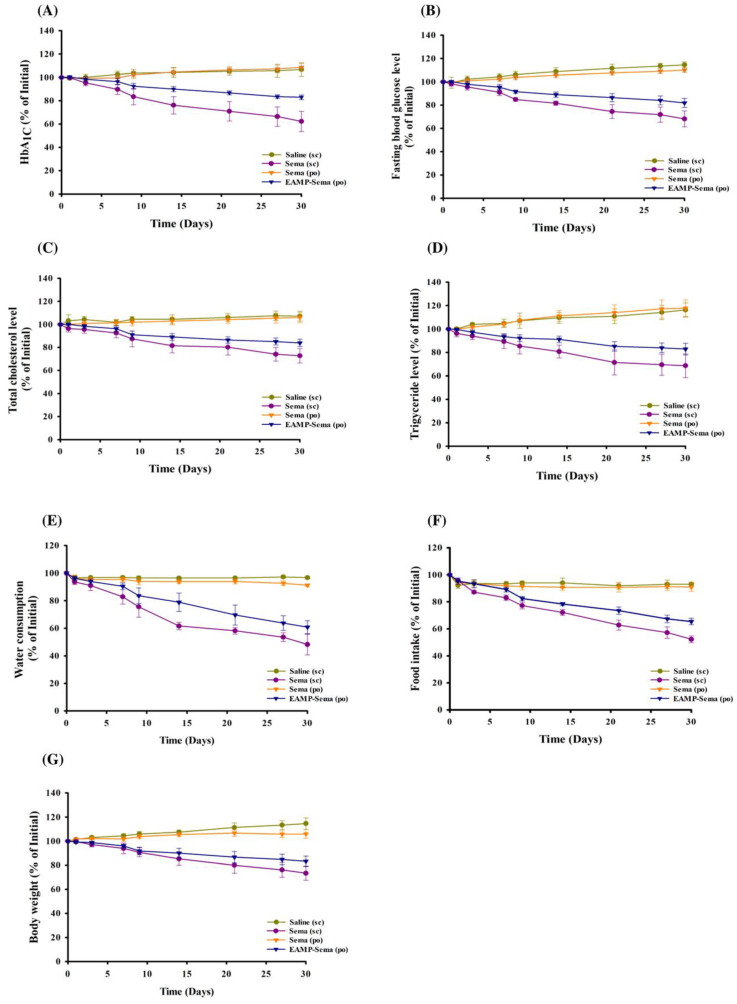
The impact of EAMP-Sema on glycemic control and weight loss of high-fat diet/streptozotocin-induced type 2 diabetic rats (mean ± SD, *n* = 6). Changes in various physiological parameters were assessed daily for 30 days: (**A**) glycated hemoglobin (HbA1c), (**B**) fasting blood glucose, (**C**) TC, (**D**) TG, (**E**) food intake, (**F**) water consumption, and (**G**) body weight. The administered doses were equivalent to 0.4 and 8 mg/kg semaglutide for subcutaneous injection and oral administration, respectively.

To assess the utility of EAMP-Sema as an oral formulation, the anti-diabetic and anti-obesity effects of orally administered EAMP-Sema were evaluated in type 2 diabetic rats and compared with those from SC injection and free semaglutide solution. As summarized in Figure 6, the SC injection of semaglutide significantly improved glycemic control and reduced body weight. These results are comparable to previous findings [37,38,39]. Although each study adopted different animal models and dosing regimens, previous studies showed similar patterns of blood glucose control, a reduction in food intake, and weight loss after semaglutide treatment compared with our observations.

After oral administration, EAMP-Sema also exhibited significant therapeutic effects in terms of glycemic control and body weight loss, while oral administration of free semaglutide did not yield comparable benefits. For the estimation of glycemic control, glycosylated hemoglobin (HbA1c) and fasting blood glucose levels were monitored over the 30-day treatment period. In the orally administered EAMP-Sema group, both HbA1c and fasting blood glucose levels gradually decreased over the treatment period, reaching around 83% and 82% of the initial levels, respectively, at the end of the study (Figure 6A,B). The plasma levels of TC and TG were also monitored over the treatment period, as glycemic control is affected by the plasma lipid profile [40,41]. As shown in Figure 6C,D, EAMP-Sema administration could reduce TC and TG levels, reaching around 84% and 83% of the initial levels, respectively.

Semaglutide can also decrease food intake by reducing appetite and food avidity, thereby improving control over eating and body weight loss [42]. Therefore, the effects of EAMP-Sema on obesity symptoms were evaluated in type 2 diabetic rats. As shown in Figure 6E–G, orally administered EAMP-Sema significantly suppressed food and water intake, thus decreasing body weight. The oral efficacy of semaglutide via EAMP-Sema may be explained by several factors. First, the surface coating of nanoparticles with a pH-dependent polymer could minimize the drug exposure to a harsh gastric environment, thereby delivering more intact drugs to the colon with low enzyme activity. In addition, the AMP-based core nanocomplex could facilitate transcellular and paracellular drug uptake. These hypothesis are also supported by the results from the in vitro drug release and cellular uptake studies of EAMP-Sema in Section 3.2 and Section 3.4. In contrast, oral administration of free semaglutide failed to show comparable benefits, which may be due to the extensive degradation of peptide drugs in the GI tract.

Although the subcutaneous injection of semaglutide exhibited greater therapeutic effects in terms of all tested physiological parameters, orally administered EAMP-Sema could significantly improve glycemic control and reduce body weight, thus displaying its potential as an oral formulation of semaglutide. Considering that orally administered protein drugs often suffer from GI instability and low intestinal absorption, AMP-based nanocomplex may provide a promising oral delivery strategy for various protein drugs. To expand the clinical application of the AMP-based nanocomplex, a more extensive in vivo safety evaluation of AMP clay is necessary to meet the regulatory requirements.

## 4. Conclusions

EAMP-Sema was fabricated as spherical nanoparticles with an average size of 318 ± 1.23 nm and entrapment efficiency of 92.5%. The secondary structure of semaglutide encapsulated in EAMP-Sema was effectively protected from exposure to simulated gastric and intestinal fluids containing proteases. Furthermore, EAMP-Sema could minimize drug release in the stomach and significantly improve cellular drug uptake. Consequently, EAMP-Sema significantly improved the oral efficacy of semaglutide, thus improving glycemic control and weight loss in type 2 diabetic rats.

## Figures and Tables

**Table 1:** Characteristics of nanoparticles (mean ± SD, *n* = 3).

Formulation	Size (nm)	PDI	Zeta Potential (mV)	EE (%)
AMP—Sema	162 ± 3.63	0.272 ± 0.032	−14.7 ± 0.35	99.7 ± 0.03
EAMP—Sema	318 ± 1.23 *	0.235 ± 0.051	−23.7 ± 0.53 *	92.5 ± 0.78

*: *p* < 0.05, compared to AMP-Sema.

## Data Availability

All data relevant to the publication are included.

## Supplementary Materials

The following supporting information can be downloaded at: https://www.mdpi.com/article/10.3390/pharmaceutics16070886/s1, Figure S1. HPLC chromatograms of semaglutide released from nanoparticles after 2h incubation in simulated gastric fluids (pH 1.2). I.S.: internal standard. Figure S2. Size and surface charge of EAMP-Sema during the incubation in Hank’s Balanced Salt Solution (HBSS) at 37 °C (mean ± SD, n = 3). Figure S3. CD spectra of semaglutide released from AMP-based nanoparticles after the 3 h-incubation in Hank’s Balanced Salt Solution (HBSS).
