# Supplementary material for: Enhanced Oral Efficacy of Semaglutide via an Ionic Nanocomplex with Organometallic Phyllosilicate in Type 2 Diabetic Rats
Source: Pharmaceutics. 2024 Jun 30;16(7):886. doi: 10.3390/pharmaceutics16070886 (PMC11280289; doi:10.3390/pharmaceutics16070886)
Supplement: Supplementary file 1 [file pharmaceutics-16-00886-s001.zip › pharmaceutics-3041582-supplementary.pdf]

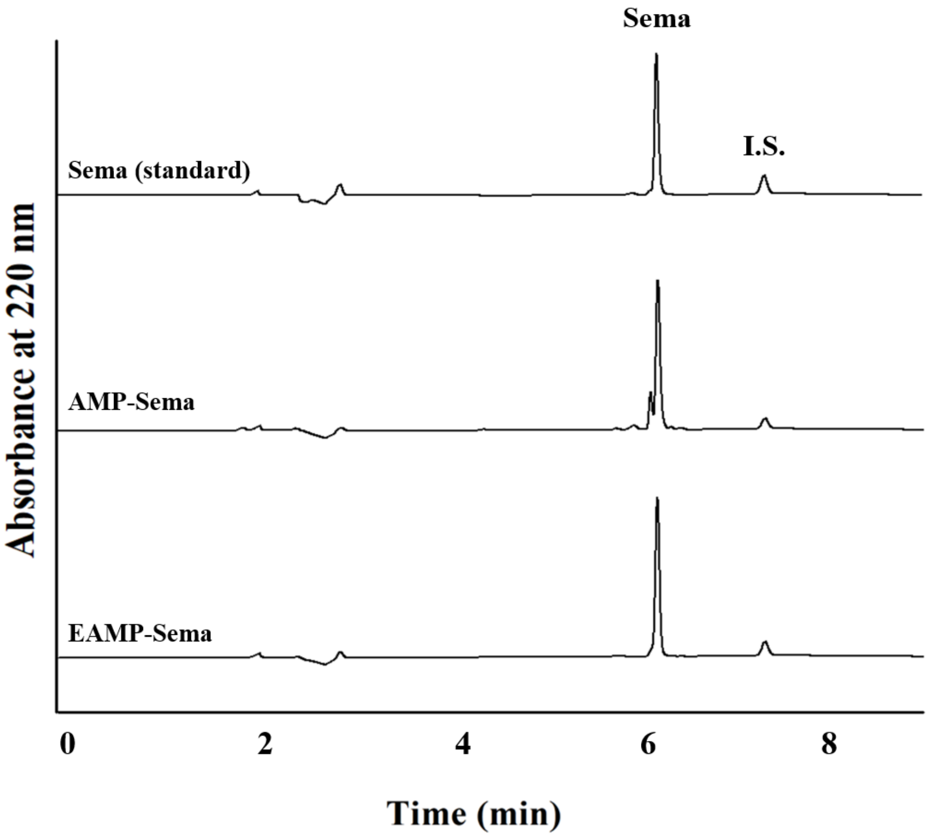

Figure S1. HPLC chromatograms of semaglutide released from nanoparticles after 2h incubation in simulated gastric fluids (pH 1.2). I.S.: internal standard.

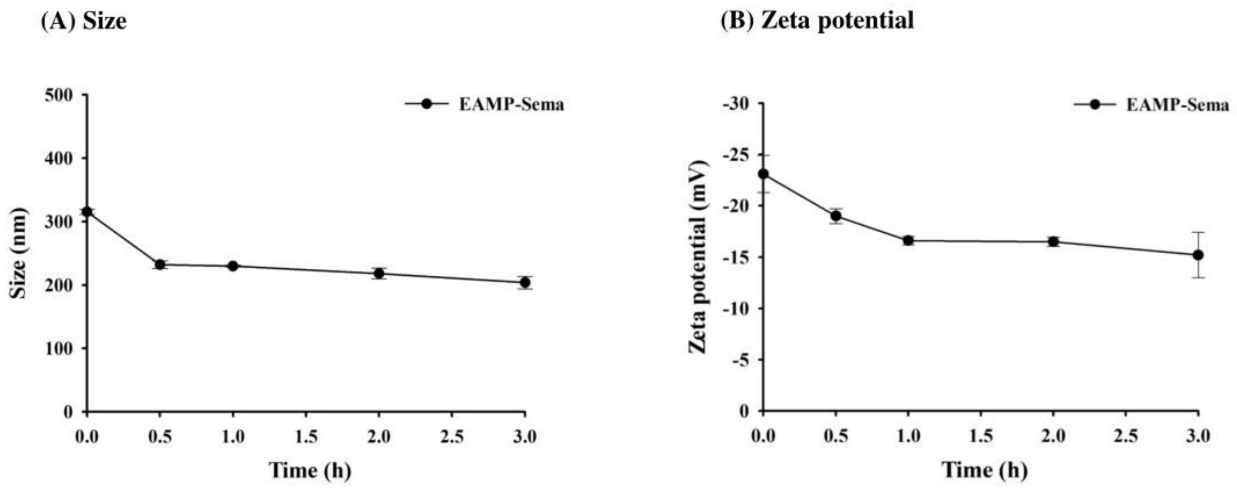

Figure S2. Size and surface charge of EAMP-Sema during the incubation in Hank's Balanced Salt Solution (HBSS) at 37 °C (mean ± SD, n = 3).

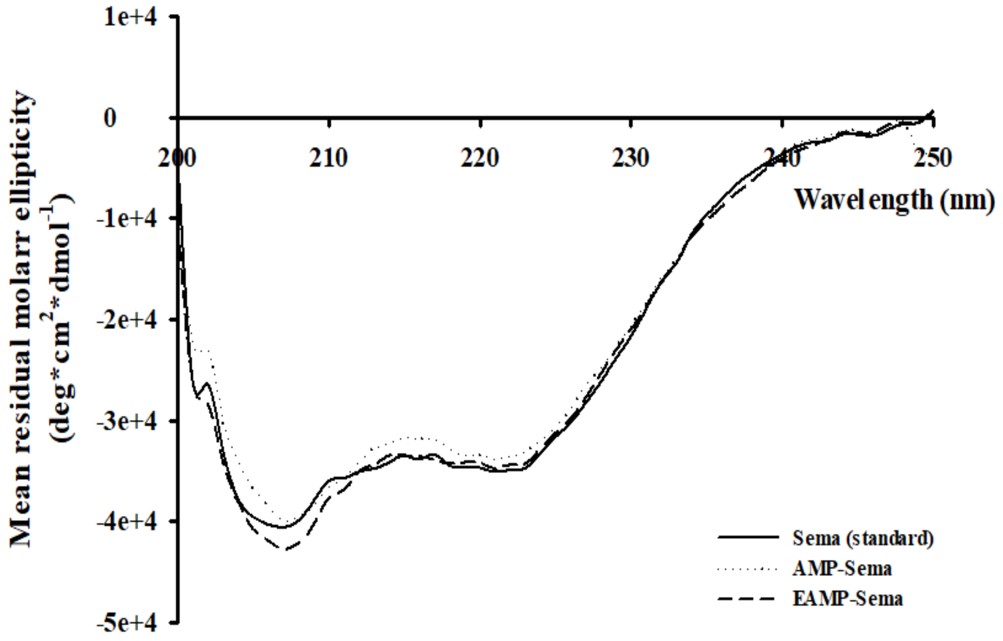

Figure S3. CD spectra of semaglutide released from AMP-based nanoparticles after the 3 h-incubation in Hank's Balanced Salt Solution (HBSS).
